# On-Chip Imaging of *Schistosoma haematobium* Eggs in Urine for Diagnosis by Computer Vision

**DOI:** 10.1371/journal.pntd.0002547

**Published:** 2013-12-05

**Authors:** Ewert Linder, Anne Grote, Sami Varjo, Nina Linder, Marianne Lebbad, Mikael Lundin, Vinod Diwan, Jari Hannuksela, Johan Lundin

**Affiliations:** 1 Department of Microbiology, Tumor and Cell Biology (MTC), Karolinska Institutet, Stockholm, Sweden; 2 Institute for Molecular Medicine Finland (FIMM), University of Helsinki, Helsinki, Finland; 3 Center for Machine Vision Research, University of Oulu, Oulu, Finland; 4 Swedish Institute for Communicable Disease Control (SMI), Solna, Sweden; 5 Global Health/IHCAR, Department of Public Health Sciences, Karolinska Institutet, Stockholm, Sweden; University of Queensland, Australia

## Abstract

**Background:**

Microscopy, being relatively easy to perform at low cost, is the universal diagnostic method for detection of most globally important parasitic infections. As quality control is hard to maintain, misdiagnosis is common, which affects both estimates of parasite burdens and patient care. Novel techniques for high-resolution imaging and image transfer over data networks may offer solutions to these problems through provision of education, quality assurance and diagnostics. Imaging can be done directly on image sensor chips, a technique possible to exploit commercially for the development of inexpensive “mini-microscopes”. Images can be transferred for analysis both visually and by computer vision both at point-of-care and at remote locations.

**Methods/Principal Findings:**

Here we describe imaging of helminth eggs using mini-microscopes constructed from webcams and mobile phone cameras. The results show that an inexpensive webcam, stripped off its optics to allow direct application of the test sample on the exposed surface of the sensor, yields images of *Schistosoma haematobium* eggs, which can be identified visually. Using a highly specific image pattern recognition algorithm, 4 out of 5 eggs observed visually could be identified.

**Conclusions/Significance:**

As proof of concept we show that an inexpensive imaging device, such as a webcam, may be easily modified into a microscope, for the detection of helminth eggs based on on-chip imaging. Furthermore, algorithms for helminth egg detection by machine vision can be generated for automated diagnostics. The results can be exploited for constructing simple imaging devices for low-cost diagnostics of urogenital schistosomiasis and other neglected tropical infectious diseases.

## Author Summary

There is a need to develop diagnostic methods for parasitic infections specifically designed for use in resource-deficient situations. Worm infections are common in many poor countries and even if repeated treatment can be arranged at low cost, diagnostics and identification of treatment failures demand resources not easily available. With the proliferation of mobile phones, data transfer networks and digital microscopy applications the stage is set for alternatives to conventional microscopy in endemic areas. Our aim was to show, as proof of concept, that it is possible to achieve point-of-care diagnostics by an inexpensive mini-microscope for direct visualization on a display and remote diagnostics by computer vision. The results show that parasitic worm eggs can be recognized by on-chip imaging using a webcam stripped off the optics. Images of eggs from the blood fluke *S. haematobium* present in urine of an infected patient could be interpreted visually and by computer vision. The method offers both an inexpensive alternative to conventional microscopy and diagnostic assistance by computer vision.

## Introduction

Microbiological diagnostics at the basic levels of the health care system has to meet the challenges of harsh environmental conditions, inadequately trained personnel and difficulties to maintain routines for quality assurance. There is a widespread failure to understand that diagnosis is essential to the prevention and treatment of disease [1] A major problem is that methods developed in well-equipped laboratories are difficult to maintain due to lack of resources [2], [3]. The methodological requirements differ depending on the purpose, diagnostics, epidemiology, effect of intervention etc. [4], [5]. Effective control measures put the focus on diagnostics, which needs to be adapted to the stage of control.

The number of countries reporting on schistosomiasis treatment increased from 17 in 2008 to 21 in 2009 but the number of people treated for schistosomiasis in 2009 is only 8.2% of the estimated number of people infected [6]. Even more disturbing is the calculation that fewer than 5% of the infected population is receiving antischistosomal treatment and the conclusion that we may be facing “one of the first great failures of the global health decade” that began in 2000 [7].

The focus is increasingly on diagnostics of chronic multiple parasitic infections affecting populations in poor rural areas [8], [9]. There is a definite shift from morbidity control to transmission control after long-term treatment campaigns [10], [11]. This underlines the need for monitoring tools. With decreasing transmission rates, factors related to chronic infection - not parasite load - become important.

Diagnostics of urogenital schistosomiasis based on the presence of blood in urine has been successfully used as a low-cost method in a high prevalence situation [12], but such indirect methods become less useful in low prevalence and low intensity situations [13]. With decreasing endemicity the requirement for high specificity becomes more and more important – and increasingly difficult to maintain. Flawed information becomes a growing problem, which may have an unexpected impact on the fundamentals of a control effort. A large-scale commitment to eliminate major neglected infectious diseases by the end of the decade, the “London Declaration on Neglected Tropical Diseases” [14] will require reliable tools for diagnostics and monitoring.

It is obvious that recent developments are going to have an impact on our possibilities to perform diagnostics even under difficult conditions in poor endemic regions. These developments affect information transfer and analysis but also novel tools for obtaining basic information.

Digital web-based microscopy over the Internet offers the possibility not only for education and quality assurance [15], a central computer may also serve as a diagnostic unit as mobile imaging devices such as mobile phones can bring microscopy in contact with diagnostics at a distance. The interpretation of an image can be performed by an expert or even by crowd-sourcing diagnostics to non-experts [16] located essentially anywhere. It is the basis for much of telemedicine and used in radiology, cardiology etc. The use of a mobile phone to transfer a microscope image as described by Frean [17], has an unexploited potential - as shown e.g. by Zimic [18] - considering the proliferation of mobile networks and the possibility of integrating various types of health care related information [19].

Novel tools are emerging for producing high magnification images. By placing a small ball lens over the mobile phone camera lens [20] or by placing objects in close proximity to the surface of a sensor chip, “on-chip imaging” [21]. These methods have the potential to revolutionize diagnostic imaging, which today can be achieved only using a microscope.

A current limitation of the methods is that either the sharp image area is very small, as in case a ball lens is used, or the resolution is limited as in case of on-chip imaging. On-chip imaging using rather elaborate computational holographic techniques such as diffraction analysis and super-resolution by the pixel shift technique [22], [23], [24] has shown that high-resolution imaging can be performed.

In this study we wanted to test if the basic technique of imaging an object directly on the surface of an image sensor chip of a webcam or a mobile phone camera - which is so simple that it has been presented as a hobby project (http://makeprojects.com/Project/Lensless-Microscope/220/1) - can be used for the diagnostics of helminth infections. As “proof-of-principle”, analysis of webcam images was done using computer algorithms for the identification of *Schistosoma haematobium* eggs in the urine. We show that it is possible to use pattern recognition to “duplicate the abilities of human vision by electronically perceiving and understanding an image approach to image analysis” [25] in the diagnostics of urogenital schistosomiasis.

## Methods

### Ethics Statement

Patient urine and stool materials containing excreted helminth eggs were handled in accordance with the Swedish “Biobanks in Medical Care Act” (2002:297) and the “New Biobanks Act” (Swedish government Report, SOU 2010:81) stating that “….samples may be collected, stored and used for certain purposes (including research and cross-border exchanges of samples and data), with respect for the individual integrity and privacy.” (http://www.hsern.eu/index.php/news/show/sw-swedish-government-published-a-report-sou-2010-81-entitled-a-new-biobank-act) Samples were not collected specifically for this study. All human samples obtained under oral consent and anonymized were from an already-existing sample collection for education and quality assurance (“Panel för Cystor & Maskägg” at SMI).

Anonymized stool samples provided by Jürg Utzinger at the Swiss Tropical and Public Health Institute, Basel, Switzerland were collected for collaborative evaluation of diagnostics and quality assurance and approved by the ethics committees of Basel (EKBB, 377/09) and Côte d'Ivoire (reference no. 1993 MSHP/CNER; date 2010-05-10).

*Schistosoma mansoni* eggs were obtained from mice kept according to national guidelines (Swedish Board of Agriculture SJVFS 2012:26). Mice were experimentally infected to provide materials for diagnostics of human infections. The protocol was evaluated and approved by the regional ethical committee Stockholm North, Dpt. 1, Stockholm District Court (reference no. N527/11; date 2011-01-26).

### Parasite Specimens and Microscopy

Experiments reported here were performed on a urine sediment obtained by pooling urines from individuals shown to excrete *S. haematobium* eggs. The formalin fixed sediment was stored at +4°C. For on-chip experiments, aliquots of the sediment were diluted in saline to give a concentration of about 250 eggs per ml. The concentration corresponds to a 10-fold concentration of 250 eggs in 10 ml of urine allowed to sediment and then re-suspended into 1 ml. The concentration of more than 50 eggs per 10 ml is considered to reflect an infection of high intensity [26], [27]. The *S. haematobium* sample was obtained from the diagnostic parasitology laboratory of the Swedish institute for communicable disease control (SMI), Solna, Sweden. Samples containing intestinal helminth eggs (*S. mansoni, Trichuris trichiura* and *Diphyllobothrium latum*) and *Strongyloides* larvae were from standard formalin or SAF-fixed (fixative containing sodium acetate, acetic acid and formalin) human stool samples. Pooled isolated *S. mansoni* eggs used for some on-chip experiments were obtained from experimentally infected mice as previously described [28], [29].

Images of helminth eggs were captured for reference purposes using established techniques: For part of the samples we used a microscope (Leica DMRB,*Leica*, *Leitz*) equipped with a digital camera (AxioCam; *Carl Zeiss; Oberkochen, Germany*) and using image capture. Imaging software (Openlab, *Improvision; Coventry, United Kingdom*) on a desktop computer (Apple Macintosh G4 with McOS 9; *Cupertino, CA*) was used for image capture. Specimens containing *S. haematobium* eggs and *Strongyloides* larvae were digitized for web-based virtual microscopy as described before [15].

Some samples were also digitized with an automated whole slide scanner (*Pannoramic P250, 3DHistech Ltd, Budapest, Hungary*), using a 20×objective (numerical aperture 0.8) equipped with a three-CCD (charge-coupled device) digital camera (CIS 3CCD, 2 megapixel, *CIS Corporation, Tokyo, Japan*). The pixel resolution was 0.22 µm. The images were compressed with a conservative compression ratio of 1∶5 to a wavelet file format (*Enhanced Compressed Wavelet, ECW, ER Mapper, Erdas Inc, Atlanta, Georgia*) and made available for web-based virtual microscopy [15].

To enable fixation of liquid samples for the whole-slide imaging, specimens of helminth eggs were mounted and immobilized under coverslips on microscope slides with a drop of glycerin-gelatin (*Sigma-Aldrich* product GG1 aqueous slide mounting medium) at 55–60°C. Samples were scanned not only in the x and y planes, but also in different focal planes in order to generate z stacks to enable focusing in the web-based viewer.

### On-Chip Imaging, Image Transfer and Generation of Digital Images

On-chip imaging was performed essentially by placing the specimen in contact with an image sensor, which was then illuminated to produce a shadow of objects present in the specimen. The CMOS (Complementary Metal Oxide Semiconductor) sensor chip of an imaging device was made available for imaging experiments by removing the optics (see supporting information S1 and S2).

The main results reported here were obtained with the exposed sensor of a low cost webcam (*Live! Cam Sync; (VFO520, 640×480 pixel, Creative Technology Ltd.* Singapore, sold by *Clas Ohlson Co*; Insjön, Sweden as *Webbkamera*, product 38-3612 for 99,00 SEK (i.e. approximately 11€) with a calculated pixel size of 3.658 µm, in which the sensor was covered with a protective glass at a level allowing direct contact with microscope slides and resolution slide (see below). In reality, the sensor area is smaller than the cover glass and thus pixel size is smaller if calculated based on images obtained (see results). We could not test the effect of bringing objects closer to the surface of this particular actual sensor as we were unable to remove the protective covering glass without causing damage to the sensor surface.

Depending on the physical appearance of the exposed sensor, additional modified imaging devices were used in experiments related to specific issues, such as the effect of image sensor pixel size on image resolution and construction of a chamber on top of the image sensor for the analysis of fluid samples. The image sensors were surrounded by protruding components of the camera circuit board, which prevented positioning of a flat microscope glass slide directly on top of the sensor chip surface. In such cases a drop of the sample was placed directly in contact with the surface of the sensor. This was done after protecting the components of the circuit board from exposure to fluid using silicone or acrylate polymer. Such on-chip experiments were performed with the exposed sensor of another webcam (“Venus” USB 2.0 PC Camera, *Vimicro Corporation; Beijing, China*, sold by *Clas Ohlson Co*; Insjön, Sweden as *Webbkamera*, product 38-4068).

Some experiments were performed using the exposed 8 megapixel (3624×2448 pixels; pixel size 1.75 µm) sensor of mobile phone after removal of the thick protective glass and replacing it with a piece of coverslip with a thickness of 0.1 mm (Sony Ericsson C905, Sony, Japan).

Superior resolution was obtained using the exposed sensor of a mobile phone (Nokia E71, 3.2 megapixel; 2048×1536 pixels, pixel size 1.75 µm, Nokia, Finland) camera. For the experiments, replacement camera modules (n = 60) were acquired for the mobile phone (E71 Camera w/Flex Ribbon; *eBay*, *Unclemartin*; China). The sensor surface was hard to access and it was difficult to protect the surrounding circuit board components from damage caused by fluid samples. As the image sensor of this particular camera module was not protected by a cover glass it was easily damaged by the drying urine sample, which became attached to the microlens polymers on the sensor surface. Thus a new camera had to be installed for each experiment.

In some modifications, a chamber consisting of a plastic test tube was fitted above the image sensor: A rectangular hole corresponding to the size of the sensor was cut in the lid of the tube and fixed to the circuit board with silicone as described above. A sedimentation chamber was obtained by attaching a test-tube to the lid. Sedimentation was allowed to take place by inverting the test tube to allow particles to sediment onto the sensor surface. The test tube with supernatant was then removed and replaced with a light source.

In all on-chip imaging experiments the resolution of images depended on the intensity, the size of the light source and the degree of collimation of light hitting the sensor. Near collimated light was obtained placing a small LED light at a distance of about 20 cm from the sensor. The distance from the light source could be decreased to about 25 mm using a plano-convex lens (radius curvature 12.7 mm, diameter 25.4 mm BK 7 KPX043, *Newport Corporation*; Irvine, CA, United States of America). As an alternative to LED light, we used indirect daylight from a 1 mm plastic monofilament core 2.2 mm diameter fibre optic cable (*HARTING*; Sibiu, România, purchased from *Elfa Distrelec AB*; Solna, Sweden).

### Size Markers and Resolution

Resolution was measured using an optical resolution slide (*NBS USAF 1951 Test chart – R70 TN8 6HA. Pyser-SGI*; Fircroft Way, Edenbridge, United Kingdom). The square glass slide was cut to a width of 25 mm in order to fit in close approximation to the exposed sensor chip of the webcam *Live! Cam Sync*.

Calibration beads of similar size as helminth eggs were polydisperse glass particle standards (refractive Index 1.51–1.52) for image analysis calibration with a range of 50–350 µm (*WhitehouseScientific Co*.; Waverton, Chester, CH3 7PB, United Kingdom, http://www.whitehousescientific.com/)

### Image-Capture and Transfer

Image-capture and transfer was done using the camera imaging software provided by the manufacturer. Still images 640×480 pixels (VGA resolution) of *S. haematobium* eggs were used to create algorithms for computer vision (see supporting information S3 and below).

### Image Recognition and Computer Algorithms

The purpose was to identify, by computer vision, *S. haematobium* eggs in on-chip images of urine sediment obtained with the modified lensless webcam (Live! Cam Sync). Image analysis algorithms were used to detect *S. haematobium* eggs in images. Images with eggs detected by the observer were classified as positive samples. (see Supporting Information S4, Algorithms for the detection of *S. haematobium* eggs by computer vision).

To develop the detection method (Algorithm 1), 243 images were used for training a parametric model. Images were *preprocessed* to normalize brightness differences and to enhance contrast [30]. The preprocessed images were thresholded based on grey values [31]. On the thresholded images, regions of interest (ROIs) were generated based on morphological methods [32]: the images were morphologically opened in order to remove small structures. Too large and too small blobs (binary large objects, [31]) were eliminated. The ROIs were classified into positive (eggs) and negative (no eggs) based on the area, shape and contrast of regions in the original image. As shape features, eccentricity and major and minor axis were used. Also, pairs of blobs within a certain distance from each other were combined to one egg hypothesis.

The parameters for the grey value threshold and for the features to classify the ROIs were derived from 660 manually labeled eggs in 243 training images. A second set of 545 labeled eggs in 119 images with was then used for testing the detection method.

The initial results of image analysis based on the Algorithm 1 described above gave results (see below) which warranted further image analysis studies using a more advanced algorithm (Algorithm 2) where images are processed by a sequence of classifiers each stage rejecting false positive samples passed through the previous stages. (See S3) The Haar-feature based cascade classifier [33] with 45 stages containing a total of 454 weak classifiers was trained using 500 cropped egg images. 400 of these were the confirmed detections from the first classification method. The training set was extended by 100 images, which were generated by applying small distortions of randomly selected cropped images. Ten thousand negative ROIs were obtained from images of urine sediment with no eggs present.

Due to the limited number of samples, the classifier was tested using seventy-five synthetically generated images where egg images were rotated and added to background. The size variation of detections was limited between ±15% of the expected egg size. (S. Varjo and J. Hannuksela: A Mobile Imaging System for Medical Diagnostics, Proc. Advanced Concepts for Intelligent Vision Systems (ACIVS 2013), Poznan, Poland, due to appear in volume 8192 of the Lecture Notes in Computer Science series.)

### Statistical Analysis

Sensitivity (recall/completeness) was calculated as the percentage of true positive (TP) divided by true positives and false negatives (FN). Positive predictive value (precision/correctness) was calculated as the percentage of true positives divided by true positives and false positives (FP).

## Results

### On-Chip Imaging and Visual Identification of Schistosome Eggs

**Figure 1:**
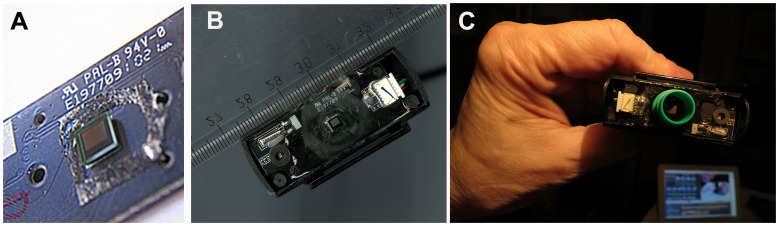
The webcam image sensor (*Live! Cam Sync*, Creative Labs) is located so that it is accessible to both fluid samples and specimens on microscope slides mounted under a coverslip. The sensor exposed after removal of optics (A). The webcam after exposure of the sensor and protecting the surrounding with polyacrylamide resin (B) and after attaching a screw-cap (with an opening cut out for the sensor) of a 10 ml plastic centrifuge tube to form a sedimentation chamber (C).

*S. haematobium* eggs could be recognized in on-chip images obtained using a simple modified webcam (Figure 1; Supporting information S1, S2 and S3). Samples on microscope slides or in liquid form could be placed directly or pipetted on the exposed sensor (Figure 1A) after protecting surrounding components with acrylic resin or silicone (Figure 1B). A chamber could easily be fitted on top of the sensor, e.g., using a pierced test tube lid (Figure 1C) and the inverted test tube could function as a sedimentation chamber. After removing the test tube, it could be replaced with an appropriate light source (see below).

### Resolution of On-Chip Images

**Figure 2:**
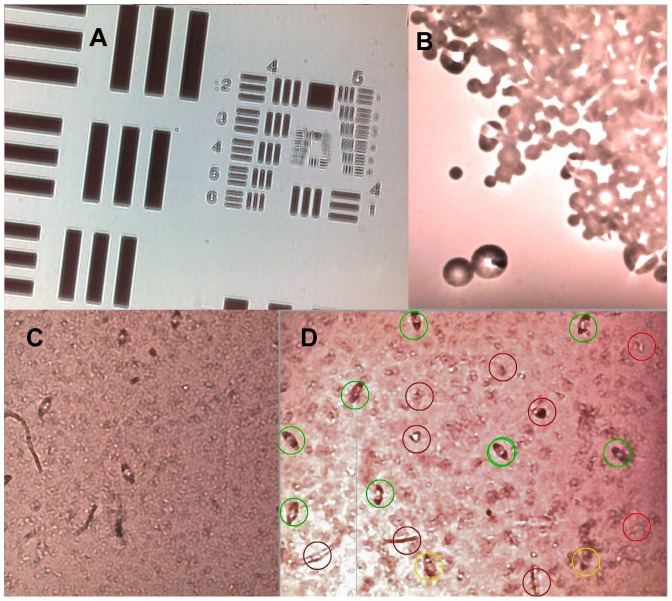
On-chip image of calibration slide *1951 USAF resolution test* chart (A), which shows resolution of 45,3 line pairs (group 5, element 4) was obtained using the lookup table (http://en.wikipedia.org/wiki/1951_USAF_resolution_test_chart). The length of element 1 in group 4 is 0. 15625 mm. The line width resolved is about 12 µm. (http://www.efg2.com/Lab/ImageProcessing/TestTargets/#Microcopy). On-chip image of calibration beads 50 to 350 µm in diameter (B). Still image obtained by pipetting urine sediment containing schistosome eggs from a patient with urinary schistosomiasis on top of an exposed, lensless webcam image sensor (C) and image used in training set to generate algorithm for computer vision (D). Green circles surround structures identified as “eggs”, red circles “not eggs” and yellow “uncertain/questionable”.

After trimming the edges of the glass supported *USAF 1951* resolution chart glass slide to 2 cm width with a glass cutter it could be positioned onto the exposed webcam image sensor. The sensors of the other devices tested were inaccessible to the resolution test slide due to components of the circuit board protruding above the sensor level. The maximum resolution of on-chip images seen on this webcam was about 40 lines per mm (group number 5, element 3 or 4 (Figure 2A). The length of schistosome eggs equaled roughly that of the line length of the first element of group 4 in the *USAF 1951* resolution chart, which is 0.15625 mm. The sample field of view dimensions correspond to the dimensions of the sensor size, which is 2.341×1.756 mm, i.e. approximately 4.11 mm^2^. As the sensor has 640×480 pixels, the calculated pixel size is 3.658 µm. Figure 2B shows on-chip image of 50–350 µm calibration beads.

The images of *S. haematobium* eggs obtained by on-chip imaging (Figure 2C and supportive information S3) were of low resolution as compared to microscope images. For reference, see scanned slide “*Schistosoma haematobium*”, in the virtual webmicroscope (fimm.webmicroscope.net/Research/Momic/helmintex). However, it was possible to use such images obtained by on-chip imaging for the development of a computer algorithm (see below).

It was necessary to adjust the amount of light directed towards the sensor. In fact adjusting the amount of light was necessary for obtaining an image. When a LED light source at a distance of about 10 cm from the sensor chip surface was used together with a pin-hole aperture of 2 mm diameter, sufficient light was obtained.

### Identification of *S. haematobium* Eggs by Computer Vision

**Figure 3:**
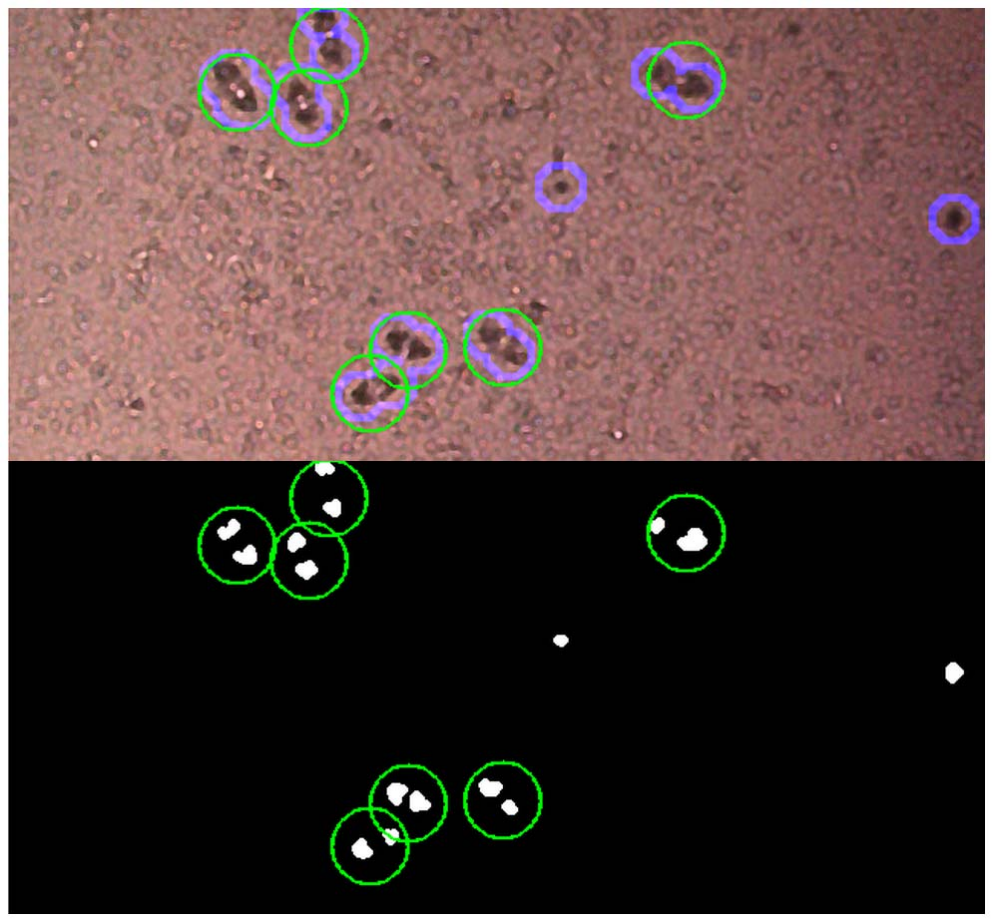
Regions of interest. Green circles mark the positions of manually labeled eggs, the light blue ones mark positions of parasites detected by computer vision.

**Figure 4:**
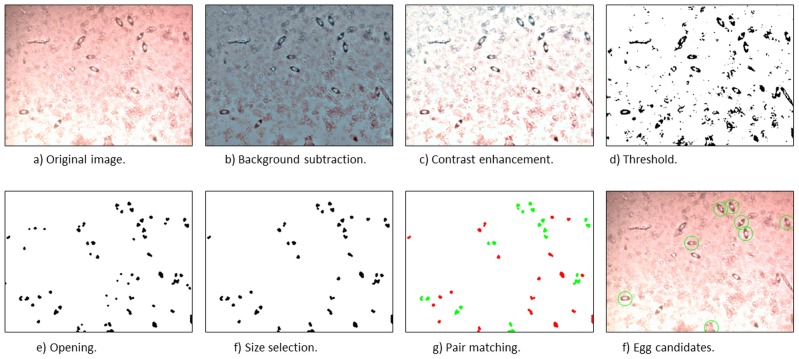
Image processing algorithm developed for the identification *of Schistosoma haematobium* eggs in urine sediment based on image capture on-chip using simple webcam. Green circle marks an egg candidate; a light blue circle marks a doubtful egg candidate. Sequential stages of image processing and analysis yielded an algorithm capable of identifying eggs in the on-chip images.

For the parameter tuning of the computer vision classifier, 660 eggs in the 243 *training images* were annotated as certain eggs (egg identified without doubt; n = 564) uncertain eggs (may be an egg; n = 96) or negative (not an egg) (Figure 2D). Five hundred sixty four were labeled “positive” and 96 were labeled “uncertain”. After training, the approach was tested on a second image set consisting of 119 *test images*, which were taken from a new sample at a later date. A total of 545 parasite eggs were manually labeled; 414 certain and 131 uncertain eggs and the object co-ordinates in the image used as reference in the evaluation of the computer vision algorithm (Figure 3 and 4).

When the performance of the algorithm (Algorithm 1) in detecting individual eggs (detection at the object level) was calculated, we obtained a sensitivity (recall/completeness) of 26% and positive predictive value (precision/correctness) of 91%.

Using the more complex cascade classifier involving 45 stages (Algorithm 2), Using the more complex cascade classifier, the sensitivity was 71% and positive predictive value 79% calculated from detections in 75 test image pairs, each image pair containing an image with a single egg and its companion image from which the egg had been replaced by background. (Sensitivity = TP/(TP+FN) = 53/(53+22) = 0.7066→70,7%), positive predictive value = TP/(TP+FP) = 53/(53+14) = 0.7910→79.1%.) (TP = true positive; FP = false positive; FN = false negative; TN = true negative).

The calculated high specificity was based on the large number of true negative images used for generating the cascade classifier (with 10000 true negative samples the specificity - TN/(FP+TN) - approached 100%.

When the performance of Algorithms 1 and 2 was compared using the same set of 75 images the difference in precision and recall was evident, but less pronounced (see supporting information S4 and S5).

### On-Chip Images of Helminth Eggs and Larvae in Stool Samples

**Figure 5:**
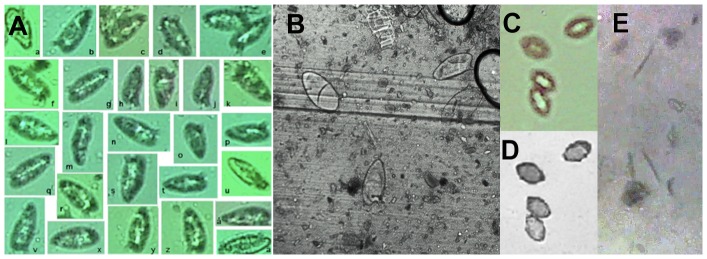
On-chip contact images of helminth eggs and larvae in stool samples. (A) Individual *Schistosoma mansoni* eggs in stool sample from experimentally infected mouse from on-chip image obtained with the exposed sensor of sensor of a mobile phone (Sony-Ericsson C905). Note that the lateral spine can be seen in some eggs. In (B) *S. mansoni* eggs are seen in an ordinary microscope using 20×objective (Leica DMRB with AxioCam digital camera). *Trichuris trichiura* eggs are seen in images obtained on-chip (C) and under conventional microscope (D) microscope using 10×objective. On-chip images were obtained with *S. mansoni*. *Strongyloides* larvae (E) by on-chip imaging using a webcam sensor (*Live! Cam Sync*, Creative Labs).

On-chip imaging experiments performed using stool samples containing various helminth eggs showed that eggs from helminths of different species could be distinguished from each other. Morphological features such as the lateral spine of *S. mansoni* eggs could be identified with certainty in some eggs (Figure 5A). The shape of *S. mansoni* eggs was clearly distinct from that of, e.g., *T. trichiura* eggs. (Figure 5C), and *Strongyloides* larvae were visible both in still images (Figure 5E) and in video recordings (see supporting information S6).

### Further Developments of On-Chip Imaging: Improved Resolution and Motion Detection

**Figure 6:**
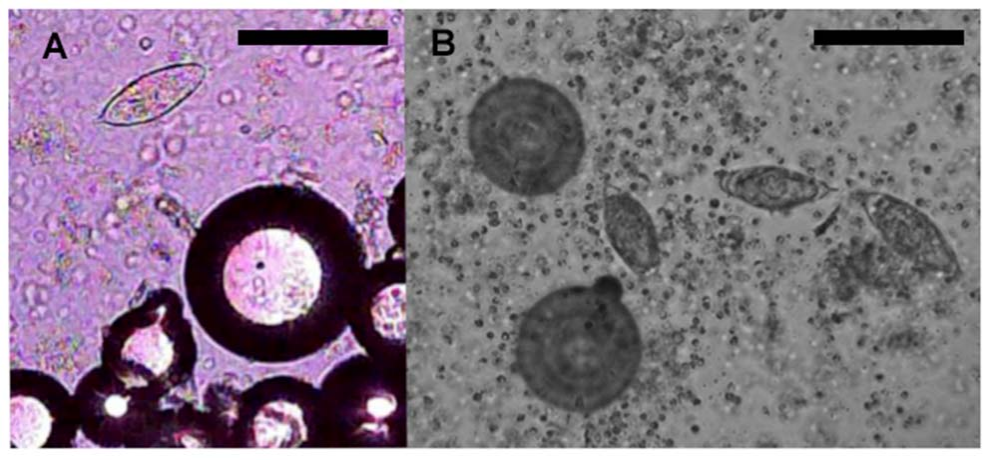
*Schistosoma haematobium* egg and calibration beads seen in on-chip image obtained with a mobile phone camera (Nokia E71) (A) compared to a similar area seen in a conventional light microscope using 10×objective (B). Note effect of absent optics in on-chip imaging; accentuated dark margins of both the egg and marker beads in on-chip image as compared to image obtained with microscope. Beads are 50 to 350 µm in diameter. Bars are 10×100 pixels.

On-chip images obtained with the webcam and Ericsson mobile phone sensors had a relatively poor resolution in comparison with images obtained by conventional microscopy (Figure 5B). However, despite technical problems due to the inaccessibility of the exposed image sensor (see above), we were able to obtain the highest resolution on-chip images with one of the mobile phone cameras in which the sensor surface was not covered by protective glass (Nokia E71; Figure 6). This setup minimized the distance between the sample and the sensor surface.

On-chip imaging provides further diagnostic possibilities based on motion detection. On-chip video recordings of stool samples showed that moving *Strongyloides* larvae in fluid specimens (Figure 5E) may be identified (see supporting information S6).

## Discussion

In the present study we show that helminth eggs placed directly on the image sensor of inexpensive imaging devices can be visualized in sufficient detail to be identified on standard displays. (se presentation; supporting information S7) If a mobile phone image sensor is used, the image can be directly sent for analysis at a distance. In this study we had access only to pooled samples and no attempt was made to establish the sensitivity of egg detection on a sample level in comparison to the standard reference method, which is expressed as egg counts per 10 ml of urine [17].

Image analysis could be performed either locally or centrally. Centralized diagnostics after image transfer may be performed based not only on visual inspection, but a central computer may also perform diagnostics more or less independently based on a computer vision algorithm. Image analysis may be performed by a remote computer [15] accessible through a network of servers [34] in much the same way as pattern recognition is applied for diverse purposes such as identification of immunoelectrophoretic patterns [35] and automated facial recognition for checking the passport at border controls [36].

Field studies are necessary in order to establish the sensitivity and specificity of both visual image interpretation and computer vision. The methods need to be assessed with ordinary microscopy as reference. Visual identification of helminth eggs present in on-chip images obtained with the simple webcam was successful, but the high specificity of image analysis was linked to a sensitivity, which needs to be improved before the method can be established in real-world situations. The performance of the computer algorithms in comparison to visual identification suggested, that we need to consider variables such as variation in egg size and shape, presence of inflammatory cells, casts, bacteria etc. in the background.

To identify *S. haematobium* eggs in urine, we developed two computer algorithms. The original algorithm based on morphological features, was capable of identifying eggs correctly (precision/positive predictive value/true positive rate) only in 26% of on-chip images with a specificity of 90%. The precision of the algorithm was 71%. The second algorithm, based on cascading classifiers had a specificity approaching 100% and an improved sensitivity of 79% as compared to visual identification.

In the present study we compared computer vision to visual image interpretation (as gold standard) and generated computer algorithms with high specificity for the evaluation of algorithm sensitivities (recall rates, which depend on the rate of false negatives) and precision (which is affected by the number of false positives). The computer algorithms generated both false positive and false negative results (about one out of four eggs), which reflect the superiority of visual interpretation of images. For a real-world test the 79% sensitivity should probably be improved to above 90%.

On chip imaging as a potential field assay for automated diagnostics depends on two parameters, first, the capacity of imaging objects (helminth eggs) with sufficient resolution to permit identification. Second, diagnostics depends on correct identification of objects in the test sample.

The image quality can be determined in terms of resolution – lines per mm -as in the present study. Clearly resolution depends on several parameters in addition to pixel size of the sensor; distance from the sample to the sensor surface and a well-controlled illumination are critical for the acquisition of on-chip images suitable for computer vision. A higher resolution will improve the accuracy of image analysis.

There is a theoretical limit to the resolution, which can be achieved, set by pixel size as stated by the Nyquist-Shannon sampling theorem, stating that the maximum achievable resolution is twice the sampling frequency. Our experimental setup using the inexpensive webcam, *Live! Cam Sync*, allowed us to detect objects with a resolution of 12.41 µm. The pixel size of the webcam image sensor was calculated to be 3.7 µm and the observed resolution therefore somewhat poorer than the theoretical resolution limit of 7.4 µm. In practice the resolution is less than the theoretical limit depending on the distance of an object to the sensor surface and due to imperfect collimation of light.

The observed image resolution was much better with the Nokia E71 3.2 megapixel image sensor (2048×1536 pixels, pixel size 1.75 µm) without a protective cover glass. The 3.5 µm theoretical resolution-limit reflects the small pixel size. Further improvements of on-chip mini-microscopes are therefore within reach, since recently introduced camera sensors have a pixel size close to 1 µm. We envision that an on-chip imaging device can be incorporated as an add-on to mobile phones capable of image transfer. On-chip imaging can benefit from the proliferation of mobile phones and the expanding data communication networks may provide the necessary infrastructure for functioning communication with a central server. Thus on-chip imaging may become an integrated part of telemedicine platform based on image capture, -transfer, -analysis and feedback.

To meet the Millennium Development Goals complex political and social re-thinking is needed at different levels [2], [37]. Health care is not isolated from the social and economic life of humans and we need to understand in detail how novel tools can be integrated in point-of-care diagnostics in much the same way as novel tools for microeconomics have revolutionized the life of “the bottom billion” [38], [39]. Limitations posed by current microscopy-based diagnostics - and national surveillance systems depending upon them- need to be resolved. Especially among poor populations of the world, microscopy needs to be adequate [40]. However complex these issues are, there is a widespread opinion that telemedicine will play an increasingly important role in managing health care in affluent and resource-poor societies alike, and tele-medical solutions will without doubt contribute to democratization of the relationship between patient-physician and family [41], [42].

Numerous compact and cost-effective optical imaging platforms, “mini-microscopes” have been developed in recent years to improve access to effective and affordable healthcare [23]. In a recent study it was shown that soil transmitted helminths can be detected in images obtained with a mobile phone [43] equipped with a small ball lens, a technique shown to generate high magnification high resolution images [21].

Like other described “mini-microscopes” on-chip imaging as described in the present paper does not require any new procedures since microscopy is the ‘gold’ standard for identification of parasitic infections. Standardized methods exist for sample collection, handling and preparation [44], [45].

The on-chip image quality is subject to diffraction artifacts caused by the absence of optical components. Proposed solutions to this problem include computational reconstruction methods (partially coherent in-line holography approach) [reviewed in 23] to obtain microscope-like images. However, in the case of imaging helminth eggs, diffraction artifacts or distortions do not seem to undermine visual identification, as seen in e.g. Fig. 6A.

One big advantage of the on-chip method described here is the large field of view - a simple webcam sensor has an area of over 10 mm^2^, e.g. more than 6 times the visual field of a conventional microscope using a 20×objective and more than 10 times the field of view using a typical ball lens [23]. On-chip microscopy, even without a computer algorithm, involves examining fewer visual fields. It can alter the tiresome routine microscopy for finding and correctly identifying parasites present in low numbers - one of the major reasons for perceived low status of the method and its failure [46], [47].

Our results suggest that automated diagnostics of helminth infections for field use using a simple imaging device and appropriate algorithms are within reach. A decisive advantage of a mini-microscope such as the one we describe, may prove to be the potential of providing diagnostic support by computer vision at a distance. Furthermore, our results suggest that diagnostics based image analysis has a potential to compete with laborious conventional microscopy e.g. by providing automated motion recognition for the detection of live nematode larvae.

## Supporting Information

Supporting Information S1 Video recording showing how to expose the sensor of WEBCAM 1 (*Vimicro Corporation, Beijing*).
(ZIP)

Supporting Information S2 Video recording showing how to expose the sensor of WEBCAM 2 *(VFO520, Creative Technology Ltd. Singapore)*.
(ZIP)

Supporting Information S3 *Schistosoma haematobium* frames. The appearance of *Schistosoma haematobium* eggs in urine sediment on-chip using “The IPEE” (modified WEBCAM 2).
(ZIP)

Supporting Information S4 Details of computer vision. Description of the automatic Schistosoma haematobium detection method. Image processing algorithms developed for the identification of Schistosoma haematobium eggs in urine sediment based on image capture on-chip using simple webcam, “The IPEE”.
(DOC)

Supporting Information S5 Precision and recall using two algorithms for the detection *Schistosoma haematobium*. Comparison of algorithms for the detection of parasite eggs in images obtained by direct on-chip imaging on webcam image sensor. *Algorithm 1* based on pattern recognition and *Algorithm 2* based on a sequence of 45 classifiers, each stage rejecting false positive samples passed through the previous stages.
(TIF)

Supporting Information S6 STRONGYLOIDES ENH.m4v (movie) The appearance of *Strongyloides* larvae in stool sample by on-chip video recording using “The IPEE” (modified WEBCAM 2).
(ZIP)

Supporting Information S7 PowerPoint presentation: “webcam microscope”. The construction of a mini-microscope, “The IPEE”, from an inexpensive webcam. Some results of imaging on such a device are seen.
(ZIP)
