# Supplementary material for: On-Chip Imaging of Schistosoma haematobium Eggs in Urine for Diagnosis by Computer Vision
Source: PLoS Negl Trop Dis. 2013 Dec 5;7(12):e2547. doi: 10.1371/journal.pntd.0002547 (PMC3855048; doi:10.1371/journal.pntd.0002547)
Supplement: Supporting Information S4 — Details of computer vision. Description of the automatic Schistosoma haematobium detection method. Image processing algorithms developed for the identification of Schistosoma haematobium eggs in urine sediment based on image capture on-chip using simple webcam, “The IPEE”. (DOC) [file pntd.0002547.s004.doc]

# S1

# ALGORITHM 1. Automatic *Schistosoma haematobium* egg detection based on morphological features

Automatic image analysis is used to detect *S. haematobium* eggs in the images. The detected eggs are used to classify images as positive (infected) or negative (control). The following text describes the method which was used in the paper in detail.

First, the images are preprocessed to normalize brightness differences and to enhance the contrast between the eggs and their surroundings. After that, the contrast-enhanced images are thresholded, and regions of interest (ROIs) are found by an opening and size selection procedure. These ROIs are classified into positive (eggs) and negative (no eggs) according to shape characteristics, neighborhood relations and a contrast measure. The classified ROIs are used to decide whether an image represents an infected sample.

Two sets of images were used to develop the method. The first set comprises 243 images and was used to develop the method and train detection parameters. The second set comprised 119 images and was used for testing the method.

## Preprocessing

Many images show significant internal brightness differences. The center often is brighter than the edges, or one part is brighter than the other. These brightness variations usually resemble continuous functions underlying the image information. To remove the brightness variations, each image was first smoothed with a Gaussian filter using a very large standard deviation σ and a corresponding large kernel. In this case, σ = 30 and a kernel size of 91x91 pixels were used. The resulting heavily smoothed image represents the average brightness of each area, approximating the underlying function. This smoothed background image was subtracted from the original image to give the brightness-corrected image with local structures preserved.


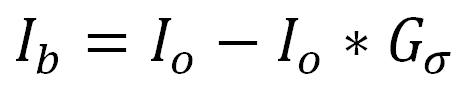
,

where *I_o_* is the original image, *G_σ_* is the Gaussian kernel with which the image is convoluted and *I_b_* is the brightness-corrected image.

In the next step, the contrast between the eggs and the surroundings was enhanced. The contrast enhancing method used here is similar to that used by Corbane et al. (2010) for ship detection in satellite imagery. It makes use of the fact that the target objects (here: the parasite eggs) are consistently darker than the background[[1]](#footnote-2). This means that their intensity is located consistently on the darker side of the maximum of the grey value histogram. To enhance contrast for the target objects, the grey values of the image between zero and the maximum of the grey value histogram are linearly stretched, mapping zero to zero and the grey level at the maximum (the mode of the histogram) to 255. Values of the original image that are larger than the histogram maximum are also mapped to 255.


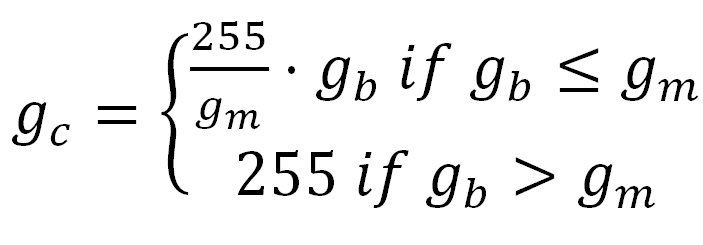
,

where *g_c_* is the contrast enhanced grey value, *g_b_* is the grey value from the brightness-corrected image, and *g_m_* is the grey value at the histogram maximum. This yields a contrast enhanced image for all values below the histogram maximum.

## Regions of interest

In the next step, *regions of interest* are detected in the preprocessed image. First, the contrast-enhanced image is thresholded using a global threshold *t* which was learned from training samples. Due to the contrast enhancing method, this threshold was relatively stable across image batches and did not need to be retrained. However, thresholding alone was not sufficient to find egg candidates as the images show many other structures of different sizes. The thresholded image was morphologically opened (Soille, 1999) using a disk of radius *r* = 4 pixels (~16µm) as structure element. The radius *r* was chosen such that it yields a disk that fits into a parasite egg after thresholding. The opening operation removes small structures and bridges between objects, it also smoothes the borders of larger structures. After opening, the image noise is greatly reduced, but there were still many blobs left which do not represent eggs. Therefore the blobs were now preselected by size. The size thresholds *a_min_* and *a_max_* are also derived from samples. Only blobs with a size between these size thresholds are selected. Small remaining noise is removed as well as large objects. The remaining blobs define a first selection of regions of interest which could conceivably represent eggs.

## Refined regions of interest

The blobs remaining after the last step include a lot of false positives. The blobs showed a wide variety of *sizes, shapes* and *spatial relations*, some of which are more closely associated than others. Many parasite eggs are indicated by two blobs of distinct size and at a distinct distance to each other. Thus blobs with a centroid[[2]](#footnote-3) distance lower than a certain maximum distance *d_max_* (derived from the training data) and higher than a minimum distance *d_min_* are counted together as one parasite egg. Blobs which constitute such a pair must have a maximum size *a_pair_* which is smaller than *a_max_*. This additional threshold is used because the two halves of one egg, which constitute a pair, obviously have a smaller area each than the whole egg.

After the pair finding, the pairs and the remaining single blobs are further evaluated separately according to shape features.

For pairs, the eccentricities of the equivalent ellipses of the blobs which constitute the pair are tested, they have to be below a maximum eccentricity. This means that the shapes of the pair blobs should not be too elongated, i.e. below a threshold *eccpair_max*.

Single blobs, which indicate parasite eggs, should be larger than blobs that are part of a pair. Therefore, only blobs with a minimum area *a_single_* considerably larger than the overall threshold *a_min_* are considered as single blob candidates. The eccentricity of the blob should be between two values *eccsingle_min* and *eccsingle_max*, in order to ensure that the shape is neither too elongated nor too round. The major and the minor axis of the equivalent ellipse also have to be within certain bounds: the major axis has to be between *lmaj_min* and *lmaj_max*, and the minor axis has to be between *lmin_max* and *lmin_min*. All these thresholds are also derived from the training data.

A spectral parameter is also used: the grey value standard deviation which is used as a measure of contrast. The standard deviation is computed in a window around the center of the blob or blob pair. The size of the window is chosen such that, approximately half the area is occupied by the parasite egg, while the rest is background. In this way, the standard deviation is a measure of the contrast between the egg and its surroundings. It has to be above a threshold *t_contrast_*.

All pairs and blobs were sorted into two groups (egg or not-egg) according to their parameter values.

## Parameter tuning

The first set of images was used for parameter tuning. In this set, clearly visible parasite eggs were labeled as “certain” and others with weaker visibility (low contrast, unusual appearance, cut off by image border) labeled as “uncertain”. The samples labeled as “certain” were used to derive parameters for the ROI detection. After normalizing and contrast enhancement, an image subset around each sample is chosen which contains the whole egg and some part of the background. For deriving the global threshold *t*, Otsu’s threshold selecting method (Otsu, 1979) was first applied to all sample subset images individually, and then the mean of these thresholds calculated.

The other parameters, which are used to classify the ROIs, were also derived from the samples. The opening operation was performed on the sample subset images, and the parameters of the blobs (size and eccentricity for pair blobs; size, eccentricity, major and minor axis for single blobs) were measured. Additionally, the maximum and minimum distances for pair blobs were measured between their centers. The standard deviation of the sample subset images was measured in the original images. For all parameters, the mean and standard deviation from the training samples are derived, and the parameter bounds are chosen in general as *mean ± standard deviation*, where upper bounds are defined as *mean + standard deviation*, and lower bounds are defined as *mean – standard deviation*.

## Results of Parameter tuning

For the parameter tuning, 660 eggs in the 243 images of the training image set were annotated as “certain” or “uncertain”. 564 were labeled ”certain” and 96 labeled “uncertain”. The threshold for the segmentation is *t* = 0.752 (grey values scaled to the interval [0;1]); the green channel was used for thresholding. Size thresholds are *a_min_* = 90 px and *a_max_* = 500 px, *a_single_* = 320 and *a_pair_* = 180. The maximum distance for blob pairs is *d_max_* = 25px, and the minimum distance is *d_min_* = 19 px. Thresholds for eccentricity are *eccpair_max* = 0.97, *eccsingle_min* = 0.35 and *eccsingle_max* = 0.970; thresholds for major and minor axis are *lmaj_max* = 34 px, *lmaj_min* = 30.5 px, *l max_min* = 17.5 px and *lmin_min* = 14.5. The threshold for the standard deviation is *tcontrast_min* = 24.

## Tests

After training, the approach was tested on a second image set consisting of 119 images, which were taken from a new sample at a later date. Parasite eggs (414 certain and 131 uncertain) were manually labeled, and their positions were used as reference in the evaluation of the method. Centroid distances are used for the mapping of reference parasite positions to detected parasite positions. Completeness is measured by searching for centroids of detected parasites inside a circle around the reference centroid position. The diameter of the circle corresponds to the length of a parasite egg. Correctness is measured by doing the same from the position of a detected parasite egg. Multiple detections are eliminated from the count.

### Test results on image level

For the evaluation on image level, images are rated positive (infected) as reference if the image contains at least one detected parasite egg; other images are negative (not infected).

Of 119 the test images, 73 were correctly classified as infected, 12 were correctly classified as not infected, none were falsely classified as infected and 34 were falsely classified as not infected. From these results, the sensitivity (sens) and the specificity (spec) are derived as statistical quality measures, defined as follows:


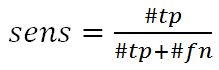

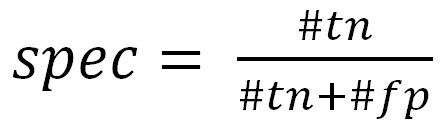


with *#tp* as the number of true positive images, *#fn* as the number of false negative images and *#fp* as the number of false positive images. The sensitivity indicates the percentage of infected samples which could be detected, in other words, how complete the detection is. The specificity indicates the percentage of not infected samples which were correctly classified as not infected. Here, the sensitivity is 68%, and the specificity is 100%.

### Test results on object level

In order to determine the quality of the detection on object level (i.e. for individual eggs) the sensitivity and the correctness (positive prediction rate) were determined. The sensitivity is defined analogously to that for the image level (see above); the correctness is defined as


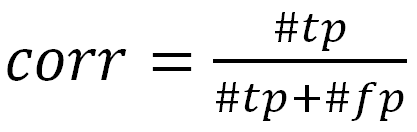


Here, *#tp* is defined as the number of true positive eggs, *#fp* is the number of false positive parasite eggs, and *#fn* (in the equation for sensitivity) is defined as the number of false negative parasite eggs. In the detection of individual eggs, a correctness of 90.6% was achieved, with a completeness of 25%. Specificity was not determined here as it is not defined for detection of objects against background.

##### References

Corbane C, Najman L, Pecoul E, Demagistri L, Petit M (2010) A complete processing chain for ship detection using optical satellite imagery. Int J Remote Sens 31(22): 5837-5854.

Otsu N A threshold selection method from gray-level histograms. IEEE T Syst Man Cyb 9(1): 62-66. 1979

Soille, P Morphological Image Analysis. Springer Verlag Berlin. 1999

# ALGORITHM 2. Cascade classifier based on Haar-like features

The cascade classifier used in the paper is a sliding window type binary detector that uses Haar-like features for weak classifiers (Viola, 2001). The basic idea here is that a small subsection of an image, the sliding window, is inspected using very simple weak classifiers which are combined first for cascade stages and the stages further for a strong binary classifier. Each cascading stage may reject the inspection window (Figure 1). Early rejection leads to improved detection speed as only a minority of features is tested. On the other hand, each passed stage increases the probability that the window contains positive detection. The image patch is labeled as a hit if all the cascading stages are passed.

**...**

reject

Stage 1

reject

Stage 2

reject

Stage 3

reject

Stage N

Figure 1: The basic structure of a cascading classifier.

##### The Haar-like features

The Haar-like features, similar to Haar-wavelets, convert an image from the pixel space to a space of feature coefficients. The Haar-like features are based on integrals of intensity (gray) values computed in given rectangular area in a search window, *r*_i_, which are weighted combinations of neighboring integrals (Lienhart , 2002). The weight ω*_i_*  in the simplest case can be plus or minus one.

*feature h = ∑*ω*_i_ RectSum( r*_i_  *)*

Figure 2 shows examples of the feature prototypes. The changes in position and size of the feature prototypes in the search window span the feature space. The search window size limits the feature space and for example with the search windows size of 22x22 pixels, that was used here, the number of used features was 60940. Summed area tables can be used to calculate these simple features very fast.

Figure 2: Examples of feature prototypes that are able to represent vertical and 45 degrees rotated edges using two integration rectangles and vertical lines using three integration rectangles.

##### Classifiers

A weak classifier consists of a Haar-like feature and a thresholding weight that separates test objects to positive and negative classes. A cascade stage is a linear combination of weighted feature values with a thresholding value resulting in binary decision. The main challenge here is to select the suitable features that differentiate the objects of interest from the background as well as the suitable weighting of the selected features and the decision boundary. These selections can be made by using well-known machine learning algorithms, such as AdaBoost (Freund, 1999). The learning algorithms basically use a set of positively and negatively labeled samples for which all the possible features are calculated and the feature that discriminates the training set best is selected. This is carried out iteratively so that the next selected feature with suitable weighing, *α*_t_, combined to the previously selected weak classifier is able to separate training set more efficiently. The training is typically carried out until the required true positive rate and false rejection rate are obtained. So a stage of a cascade classifier, *H(x)*, consist of a number, *t*, of weak classifiers *h_t_* , and a stage threshold that assigns the output of the classifier to the class label.

*H(x)* = *∑ α*_t_ *h_t_  ,*

The threshold, *Th*, may further be controlled by a polarity term, *θ*, which indicates on which side of the threshold value the positive class is:


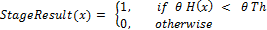


The final strong classifier is a combination of classification stages. While training the classifier stages the true positive rate is typically kept high so that no hits are missed in the cascading stages. The false rejection rate can be set relatively high value as it is improved by each cascading stage in exponential manner.

##### Testing

The training data for the classifiers came from the set of positively detected images from the first detection algorithm. An additional set of 100 images was created by applying geometrical distortions on the positive samples to slightly increase the training set. The training samples were resized to size of 22x22 pixels. This reduces computational complexity and speed up the detector.

A separate synthetic set of images with rotated positive samples added to background was used for performance testing. The negative samples came from background images without eggs.

In this work, the minimum hit rate for each cascade stage was required to be 99%. The false positive rate for each stage can be relatively high as each of the cascading stages improve the specificity by removing the false positives. While typically the maximum false alarm rate is kept at about 60%, here instead 82% was utilized to ensure training convergence with a limited number of training samples (500 positive, and 10000 negative). The selected false alarm rate yields with trained 45 cascading stages (with total of 454 weak classifiers) a theoretical false positive rate FPR = 0.82^45^ * 100% = 0.013%..

The detection hit size was limited to ±20% of the expected sample size (44x44 pixels). Also the variation for the positive hit position was allowed to differ 24 pixels from the ground truth. .

Test with 75 sample images containing single egg resulted in precision, *prec = #TP/(#TP+#FP)*, of 79.1% and sensitivity, *sens = #TP/(#TP+#FN),* of 70.7%. With 100 test images these values were 80.2 % and 65 % respectively. .

As each of the test images (640x480) containted a single positive instance of size 44x44 pixels, there remains [640-44 * 480-44] – 44x44 = 257920 negative window locations per image. This yielded specificity, *spec =* #TN/(#TN+FP) of 100% (error = 6,2 * 10^-7^).

.

Table: Classification tests

| Number of Images | True Positives (TP) | False Positives (FP) | False Negatives (FN) |
| --- | --- | --- | --- |
| 75 | 53 | 14 | 22 |
| 100 | 65 | 16 | 35 |

##### References

Paul Viola and Michael Jones “Robust Real-time Object Detection”, Proceedings of the 2001 IEEE Computer Society Conference on Computer Vision and Pattern Recognition International Journal of Computer Vision 1: 511-518. 2001

Rainer Lienhart and Jochen Maydt “An Extended Set of Haar-like Features for Rapid Object Detection”, in proceedings of the International Conference on Image Processing 2002 vol. 1, I900 – I903. 2002

Yoav Freund and Robert E. Shapiro ”A Short Introduction to Boosting”, Journal of Japanese Society of Artificial Intelligence, 14(5): 771-780. 1999

1. In Corbane’s case, the ships were brighter than the background. [↑](#footnote-ref-2)
2. geometric center; determined by calculating the mean values of the coordinates of all pixels inside the object. [↑](#footnote-ref-3)
