# Supplementary figures and images for: On-Chip Imaging of Schistosoma haematobium Eggs in Urine for Diagnosis by Computer Vision
Source: PLoS Negl Trop Dis. 2013 Dec 5;7(12):e2547. doi: 10.1371/journal.pntd.0002547 (PMC3855048; doi:10.1371/journal.pntd.0002547)

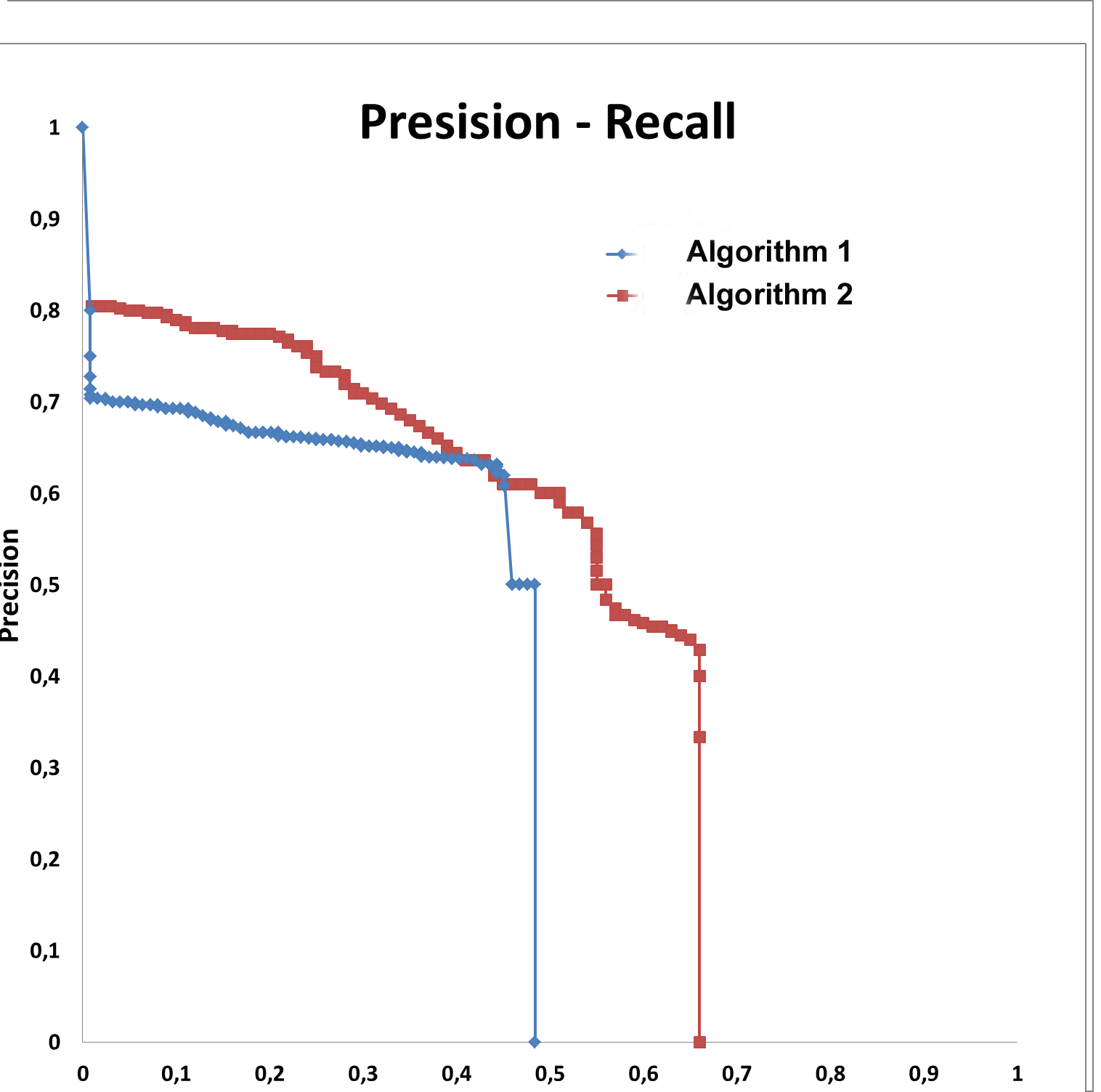

Supplement: Supporting Information S5 — Precision and recall using two algorithms for the detection Schistosoma haematobium. Comparison of algorithms for the detection of parasite eggs in images obtained by direct on-chip imaging on webcam image sensor. Algorithm 1 based on pattern recognition and Algorithm 2 based on a sequence of 45 classifiers, each stage rejecting false positive samples passed through the previous stages. (TIF) [file pntd.0002547.s005.tif]
